# CCRL2 Modulates Physiological and Pathological Angiogenesis During Retinal Development

**DOI:** 10.3389/fcell.2021.808455

**Published:** 2021-12-23

**Authors:** Cyrine Ben Dhaou, Annalisa Del Prete, Silvano Sozzani, Marc Parmentier

**Affiliations:** ^1^ WELBIO and I.R.I.B.H.M., Université Libre de Bruxelles, Brussels, Belgium; ^2^ Physiologie de La Reproduction et des Comportements, INRA Val-de-Loire UMR-85, CNRS UMR-1247, University of Tours, Tours, France; ^3^ Department of Molecular and Translational Medicine, University of Brescia and Humanitas Clinical and Research Center-IRCCS, Brescia, Italy; ^4^ Laboratory Affiliated to Istituto Pasteur Italia-Fondazione Cenci Bolognetti, Department of Molecular Medicine, Sapienza University of Rome, Rome, Italy; ^5^ IRCCS Neuromed, Pozzilli, Italy

**Keywords:** chemerin, CCRL2, CMKLR1, ChemR23, retinal angiogenesis, oxygen-induced retinopathy, G protein-coupled receptors (GPCRs)

## Abstract

Chemerin is a multifunctional protein involved in the regulation of inflammation, metabolism, and tumorigenesis. It binds to three receptors, CMKLR1, GPR1 and CCRL2. CMKLR1 is a fully functional receptor mediating most of the known activities of chemerin. CCRL2 does not seem to couple to any intracellular signaling pathway and is presently considered as an atypical receptor able to present the protein to cells expressing CMKLR1. CCRL2 is expressed by many cell types including leukocyte subsets and endothelial cells, and its expression is strongly upregulated by inflammatory stimuli. We recently reported that chemerin can negatively regulate the angiogenesis process, including during the development of the vascular network in mouse retina. The role of CCRL2 in angiogenesis was unexplored so far. In the present work, we demonstrate that mice lacking CCRL2 exhibit a lower density of vessels in the developing retina and this phenotype persists in adulthood, in a CMKLR1-dependent manner. Vascular sprouting was not affected, while vessel pruning, and endothelial cell apoptosis were increased. Pathological angiogenesis was also reduced in CCRL2^-/-^ mice in a model of oxygen-induced retinopathy. The phenotype closely mimics that of mice overexpressing chemerin, and the concentration of chemerin was found elevated in the blood of newborn mice, when the retinal vasculature develops. CCRL2 appears therefore to regulate the distribution and concentration of chemerin in organs, regulating thereby its bioactivity.

## Introduction

C-C Motif Chemokine Receptor Like (CCRL2, also known as CRAM or HCR in human and L-CCR in mouse), was first identified in 1998 as a potential chemokine receptor upregulated in murine macrophages after LPS stimulation (Fan et al., 1998; Shimada et al., 1998). CCRL2 is expressed in most leukocyte populations, including monocytes, macrophages, DCs, neutrophils, CD4^+^ and CD8^+^ T cells, B cells, NK cells, and mast cells (Brouwer et al., 2004; Oostendorp et al., 2004; Zabel et al., 2008; Otero et al., 2010). Its expression is strongly upregulated in monocytes/macrophages, DCs and neutrophils by pro-inflammatory stimuli, such as LPS, TNF-α, interferon-γ or CD40 ligand (Patel et al., 2001; Migeotte et al., 2002; Galligan et al., 2004; Hartmann et al., 2008). CCRL2 expression was also described in other cell types, particularly in inflammatory conditions, including microglial cells, astrocytes, airway epithelial cells, hepatic stellate cells, adipocytes, and endothelial cells (Oostendorp et al., 2004; Zabel et al., 2008; Monnier et al., 2012). CCRL2 shares high sequence similarities with receptors for inflammatory CC chemokines, such as CCR1, CCR2, CCR3 and CCR5 (Shimada et al., 1998) and its gene is located in the same cluster as these receptors’ genes on human chromosomal segment 3p21. However, similarly to the atypical chemokine receptors ACKR1 (DARC) and ACKR2 (D6), CCRL2 lacks the DRYLAIV motif that is essential for G protein coupling and chemotactic responses (Shimada et al., 1998).

CCRL2 was reported to bind the chemokines CCL2, CCL5, CCL7, CCL8 (Biber et al., 2003) as well as CCL19 (Leick et al., 2010), but these observations were not confirmed (Zabel et al., 2008; Del Prete et al., 2013; De Henau et al., 2016). Rather, CCRL2 was shown to bind with high affinity a non-chemokine chemoattractant factor, chemerin, without triggering any signaling (Zabel et al., 2008).

Chemerin has two other receptors, CMKLR1 (also known as chemerin_1_ or ChemR23) and GPR1 (chemerin_2_) and is so far the only commonly accepted ligand of CCRL2 (Wittamer et al., 2003; Barnea et al., 2008; Kennedy and Davenport, 2018). The three chemerin receptors have very different functional properties. CMKLR1 is a classical G_i_-coupled receptor, with full signaling capabilities through the inhibition of adenylate cyclase, and the activation of phospholipase C, PI3K/AKT and ERK1/2 pathways. Chemerin promotes also efficient *β*-arrestin recruitment and rapid internalization of this receptor. CMKLR1 is expressed in monocytes/macrophages, myeloid and plasmacytoid dendritic cells and natural killer (NK) cells, and chemerin is a potent chemoattractant factor for these cell populations. The recruitment of these leukocyte subsets can contribute to inflammation and the mounting of adaptive immune responses, but anti-inflammatory properties have also been described in several disease models (Bondue et al., 2011). Besides leukocytes, CMKLR1 is also expressed by endothelial cells and vascular smooth muscle cells, suggesting potential roles in angiogenesis and the control of vascular tone (Kennedy and Davenport, 2018). CMKLR1 mediates most of the known biological effects of chemerin, including the chemotactic activity, the modulation of glucose and lipid metabolism, and the regulation of tumorigenesis and angiogenesis.

GPR1 is instead a receptor characterized by a very weak coupling to G protein-mediated pathways, while promoting strong *β*-arrestin recruitment and internalizing robustly in response to chemerin (Barnea et al., 2008; De Henau et al., 2016). It is expressed mostly in the central nervous system, but also in skeletal and smooth muscle cells and adipocytes. Very little is known regarding the precise contribution of GPR1 to the functional activities of chemerin.

While the only accepted ligand of CCRL2 is chemerin, it does not trigger any known signaling through this receptor. In addition, CCRL2 does not recruit *β*-arrestin, and chemerin does not modify the slow basal recycling of the receptor between the plasma membrane and its intracellular stores (Zabel et al., 2008; De Henau et al., 2016; Mazzotti et al., 2017). CCRL2 appears therefore as an atypical receptor, with properties different from those of atypical chemokine receptors (ACKR) that mediate ligand scavenging and transcellular transport (Fra et al., 2003; Bachelerie et al., 2014; Bonecchi and Graham, 2016). CCRL2 was proposed to act as a chemerin binding site, and because the binding to CCRL2 and CMKLR1 involves different domains of chemerin, it has the unique ability to display chemerin at the surface of cells, thereby favouring its interaction with CMKLR1 expressed by neighbouring cells (Zabel et al., 2008). In particular, the C-terminus of chemerin, which is essential for the activation of CMKLR1, does not contribute to the interaction with CCRL2. Although unable to signal by itself, CCRL2 can therefore influence the bioactivity of chemerin by exposing the ligand and modifying the local concentration and distribution of chemerin in tissues. In this frame, expression of CCRL2 was shown to influence inflammatory states and tumorigenesis in different experimental models in mice (Monnier et al., 2012; Del Prete et al., 2019; Schioppa et al., 2020; Al Delbany et al., 2021). CCRL2 was also shown to modulate the activity of other receptors such as CXCR2, by forming heterodimers with these receptors (Del Prete et al., 2017).

As in other cell populations, CCRL2 is strongly upregulated by inflammatory stimuli in endothelial cells (Monnier et al., 2012). This upregulation contributes to the recruitment from the bloodstream of leukocytes displaying CMKLR1, particularly NK cells (Del Prete et al., 2019; Schioppa et al., 2020). However, despite the close interconnection between inflammation and angiogenesis, the role of CCRL2 in angiogenesis was not investigated so far. Chemerin was described to regulate angiogenesis in different ways. Several reports described pro-angiogenic properties of the chemerin-CMKLR1 pathway in tube formation assays using human umbilical vein endothelial cells (HUVEC) *in vitro* (Bozaoglu et al., 2010; Kaur et al., 2010) and more recently in *in vivo* models in mice (Nakamura et al., 2018). Chemerin was also reported to display anti-tumoral properties in mouse models (Zabel et al., 2008; Dubois-Vedrenne et al., 2019), and we identified rather an inhibitory effect on tumoral angiogenesis as the driving mechanism (Dubois-Vedrenne et al., 2021). The anti-angiogenic properties of chemerin were confirmed in the model of neonatal development of the mouse retinal vascular network (Ben Dhaou et al., 2021), in which chemerin favored blood vessel regression and endothelial cell apoptosis, thereby reducing the density of the network. Similar observations were made in the oxygen-induced retinopathy and hind limb ischemia models (Ben Dhaou et al., 2021). An anti-angiogenic effect of chemerin was also observed in the bead-sprouting assay using HUVECs, while we could not confirm the pro-angiogenic properties in tube-forming assays described previously (Dubois-Vedrenne et al., 2021).

Considering the properties of chemerin in the angiogenesis process, the expression of CCRL2 by endothelial cells and its known ability to modulate the bioactivity of chemerin, we investigated whether CCRL2 could influence physiological and pathological angiogenesis using the developing mouse retina as a model. We observed in CCRL2^-/-^ mice a reduction in the density of the vascular network as a consequence of vessel pruning and EC apoptosis. This phenotype recapitulates the observations made previously in mice overexpressing chemerin, and the blood concentration of this ligand was found to be elevated in CCRL2-deficient mice. This work highlights the important role of CCRL2 as a regulator of chemerin activity in physiological contexts *in vivo*.

## Materials and Methods

### Mouse Lines

C57BL/6J mice were obtained from Janvier. The CCRL2 (Otero et al., 2010) and CMKLR1 (Luangsay et al., 2009) knockout mouse lines were described previously and bred on the C57BL/6J background. Mice were housed in a specific pathogen-free (SPF) facility with environmental enrichment and unlimited access to food and water. Experiments were held on animals aged from 2 days to 10 weeks. Groups of control (+/+) and knockout (-/-) mice were made from littermates generated by crosses between heterozygous (+/-) animals. Experiments were made in accordance with European guidelines and Belgian regulations. They were approved by the local ethics committee (Commission d'Ethique du Bien-Etre Animal, CEBEA) of the ULB Medical School.

### Light and Fluorescence Microscopy

For light microscopy, eyes were collected, fixed in Davidson’s fixative for 16 h, embedded in paraffin, cut into 5 μm sections and stained with hematoxylin and eosin.

For fluorescence microscopy, eyes were collected and fixed in 4% paraformaldehyde in phosphate-buffered saline (PBS) for 20 min at 4°C (Pitulescu et al., 2010). After dissection in PBS, retinas were incubated in 10 mM Tris HCl, pH 7.4, 150 mM NaCl, 3% blocking reagent (ThermoFisher, 37,580), 0.5% Triton X-100 (TNBT) at 4°C for 16 h, then overnight at 4°C with Alexa Fluor 647-conjugated isolectin GS-IB_4_ (IsoB4, 1:500, I32450, Invitrogen) and primary antibodies diluted in TNBT. Primary antibodies were rabbit polyclonal anti-mouse collagen IV (1:400, Bio-Rad, 2150-1470), rabbit polyclonal anti-cleaved caspase 3 (1:400, Cell Signaling, 9661), rabbit monoclonal anti-ERG (1:500, Abcam, ab110639) and goat polyclonal anti-mouse ESM1 (1:100, R&D Systems, AF 1999). Retinas were washed three times in PBS and incubated with secondary antibodies (Alexa Fluor 488-conjugated donkey anti-rabbit IgG or Alexa Fluor 488-conjugated chicken anti-goat IgG) for 2 h at room temperature (RT). Retinas were washed and mounted on glass slides in FluorSave medium (Millipore, 345,789).

### Oxygen-Induced Retinopathy Model

Mice were housed in a 75% oxygen environment from P7 to P12, and back to normoxic conditions afterwards (Campochiaro and Hackett, 2003). Retinas were collected at the end of the hyperoxic period (P12) and after 5 days of normoxia (P17).

### Morphometric Analyses

Confocal images were acquired on a Zeiss LSM 780 NLO microscope (Zeiss) equipped with a Chameleon Vision II 690–1064 nm multiphoton laser (Coherent Europe). Parameters of the retinal vascular network (vessels density, total vessels length, junctions density) were analyzed by the Angiotool software (Zudaire et al., 2011) on images of IsoB4-stained retinal leaflets obtained with a 20x objective. These parameters were measured in different fields located in the remodeling or proliferative zones of the network and normalized for the surface analyzed. For each of these parameters, a data point was considered as the mean of all values obtained for a retina.

For the determination of the radial expansion of the network, images of whole retinas stained with IsoB4 were reconstructed from tiles obtained with a 20x objective, the distances separating the network front and the retinal edge from the optic disc were measured and the ratio calculated. A data point was considered as the mean of values obtained for all leaflets of a retina.

For the quantification of endothelial cells and tip cells, the retinas were stained with IsoB4 and for ESM1 and ERG, and reconstructed images (20x) of whole retinas were built. The density of ERG^+^ cells was calculated in Fiji, as well as the linear density of ESM1^+^ ERG^+^ cells along the angiogenic front of the network. A data point was considered as the mean of all values obtained for a retina.

Empty sleeves were visualized by staining endothelial cells with IsoB4 and basal membranes for ColIV. Images of whole retinas were reconstructed from tiles (20x) and analyzed in Fiji. The density of empty sleeves (ColIV^+^ IsoB4^-^) was calculated in the remodeling and proliferative zones, either in the vicinity of main arteries and first order arterial branches, or around main veins and first order vein branches. Damaged areas were excluded. For each parameter, a data point was considered as the mean of all values obtained for a retina. The number of apoptotic endothelial cells was counted in the vicinity of main arteries and first order arterial branches on retina quadrants stained with IsoB4 and for cleaved caspase three and divided by the surface.

For the oxygen-induced retinopathy (OIR) model, retinas were stained with IsoB4 and imaged at 20x. The Fiji software was used to determine the area devoid of vasculature and that of neovascular tufts, which were divided by the total surface of the retina. For the determination of vascular parameters of mouse retinas, including in the OIR model, the body weight was measured, and mice over two standard deviations from the mean were not considered.

### Immunoassays

Chemerin concentrations were assessed in the plasma of CCRL2^-/-^ and control pups, using a mouse chemerin DuoSet ELISA kit (R&D Systems, DY2325), following the manufacturer’s instructions.

### Statistics

Data are presented as mean ± SEM. Statistical significance was calculated by unpaired two-tailed Student’s t-test, using Instat or Graphpad Prism 7 softwares. For all experiments, knockout and control animals were littermates, and age-matched animals were randomly distributed into groups. The genotype and experimental conditions were not known at the time of data collection.

## Results

### CCRL2 Regulates the Density of Blood Vessels in the Developing Retina

**FIGURE 1:**
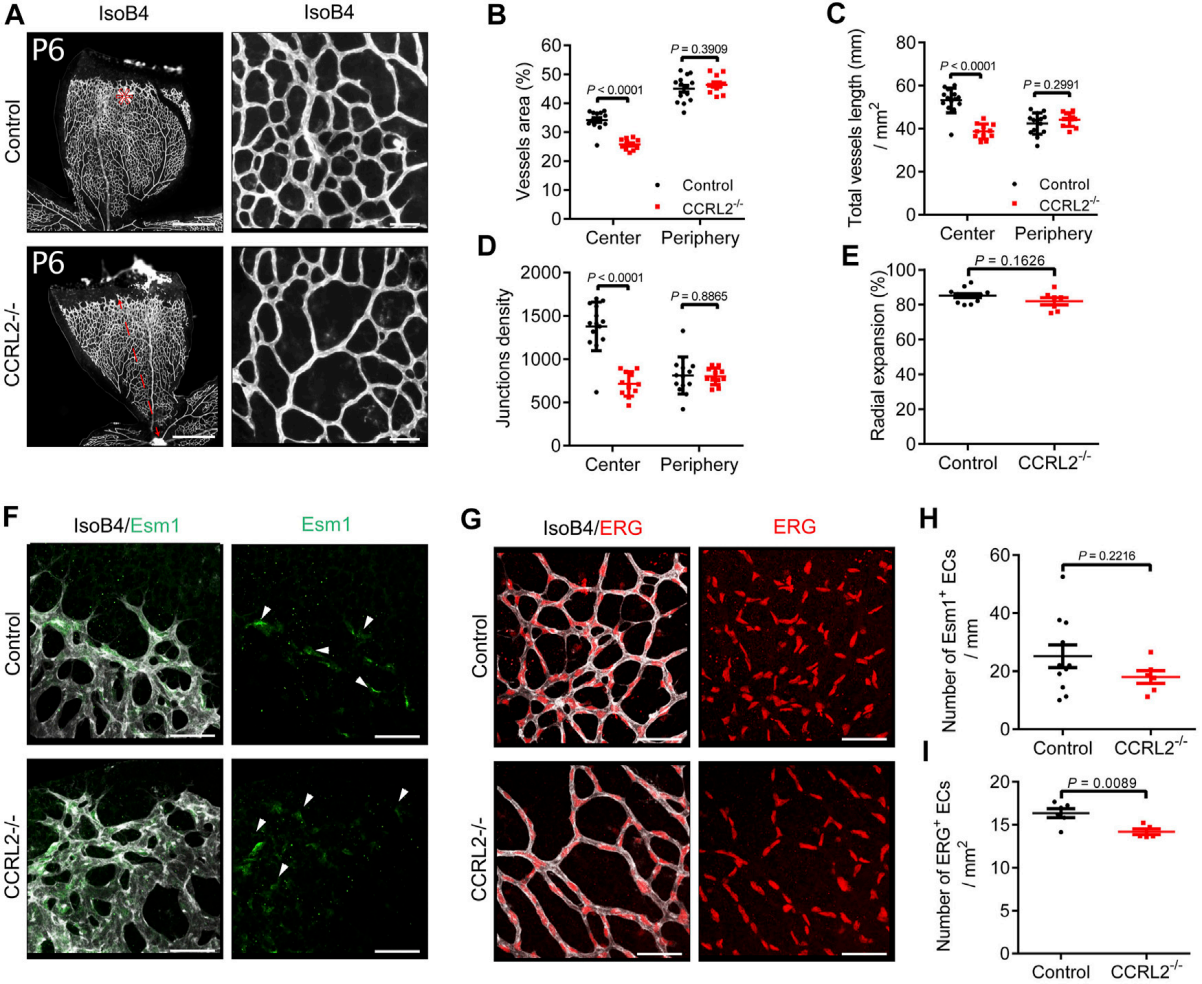
CCRL2 loss-of-function reduces the density of the vascular network in the developing mouse retina. **(A)** Retinas from CCRL2^-/-^ and control mice at post-natal day 6 (P6), stained with isolectin B4 (IsoB4). Scale bars: 500 μm in left panels, 50 μm in right panels. **(B–D)** Vessels area, total vessels length and junctions density, relative to the surface, in the angiogenic and remodeling zones of the vascular plexus (*n* = 14 for controls, *n* = 11 for CCRL2^-/-^). **(E)** Radial expansion of the vascular network (% of retinal radius) in control and CCRL2^-/-^ mice (*n* = 12 for controls, *n* = 7 for CCRL2^-/-^). **(F)** Angiogenic zone of the retinal network of P6 mice, stained with IsoB4 and for ESM1. Endothelial tip cells positive for ESM1 are indicated by white arrows. Scale bars: 50 μm. **(G)** Remodeling zone of the retinal network of P6 mice in control and CCRL2^-/-^ mice, stained with IsoB4 and for ERG. Scale bars: 50 μm. **(H)** Linear density of ESM1^+^ tip cells at the front of the vascular network in control (*n* = 11) and CCRL2^-/-^ mice (*n* = 6). **I** Density of ERG^+^ endothelial cells in the remodeling zone of the vascular plexus in the retina of control (*n* = 6) and CCRL2^-/-^ mice (*n* = 5). Mean ± SEM, 2-tailed unpaired Student’s t test. Each point represents one animal.

Considering the role played by chemerin during the development of the vascular network in the post-natal mouse retina, we investigated the potential role of the non-functional chemerin receptor, CCRL2, in the same process. This *in vivo* model of physiological angiogenesis allows to monitor the various mechanisms contributing to angiogenesis, such as endothelial cell sprouting, pruning and vessel maturation (Fruttiger, 2007). The retina of mice deficient for CCRL2 (CCRL2^-/-^) and their control littermates were analyzed at various time-points. At post-natal day 6 (P6), the density of the vascular network under development was lower in CCRL2^-/-^ mice than in control littermates (Figure 1A). The various parameters recorded, including the surface of vessels (Figure 1B), cumulative vessels length (Figure 1C) and number of branch points (Figure 1D), normalized for the analyzed area, were significantly lower in the central part of the network in CCRL2^-/-^ retina. The same parameters were however not affected in the peripheral part of the network, behind the tip cell zone (Figures 1A–D, asterisk).

The progression of the network from the central artery and vein to the periphery of the retina was not affected CCRL2^-/-^ mice. Indeed, the distance separating the superficial vascular front from the central artery was unaffected at P6 (Figure 1A, red line, and Figure 1E). Also, no changes were seen in the linear density of tip cells, stained for the endothelial cell-specific molecule 1 (ESM1) marker (del Toro et al., 2010; Rocha et al., 2014) along the network front (Figures 1F,H). However, the density of endothelial cells, determined after nuclear staining for the transcription factor ERG, was lower in the central part of the plexus (Figures 1G,I). These observations suggested that CCRL2 loss-of-function did not affect EC sprouting, but rather increased vessel regression during the remodeling phase of vascular development, a phenotype similar to that observed previously in mice overexpressing chemerin (Ben Dhaou et al., 2021).

### CCRL2 Loss-Of-Function Favors Vessel Regression and Endothelial Cell Apoptosis

**FIGURE 2:**
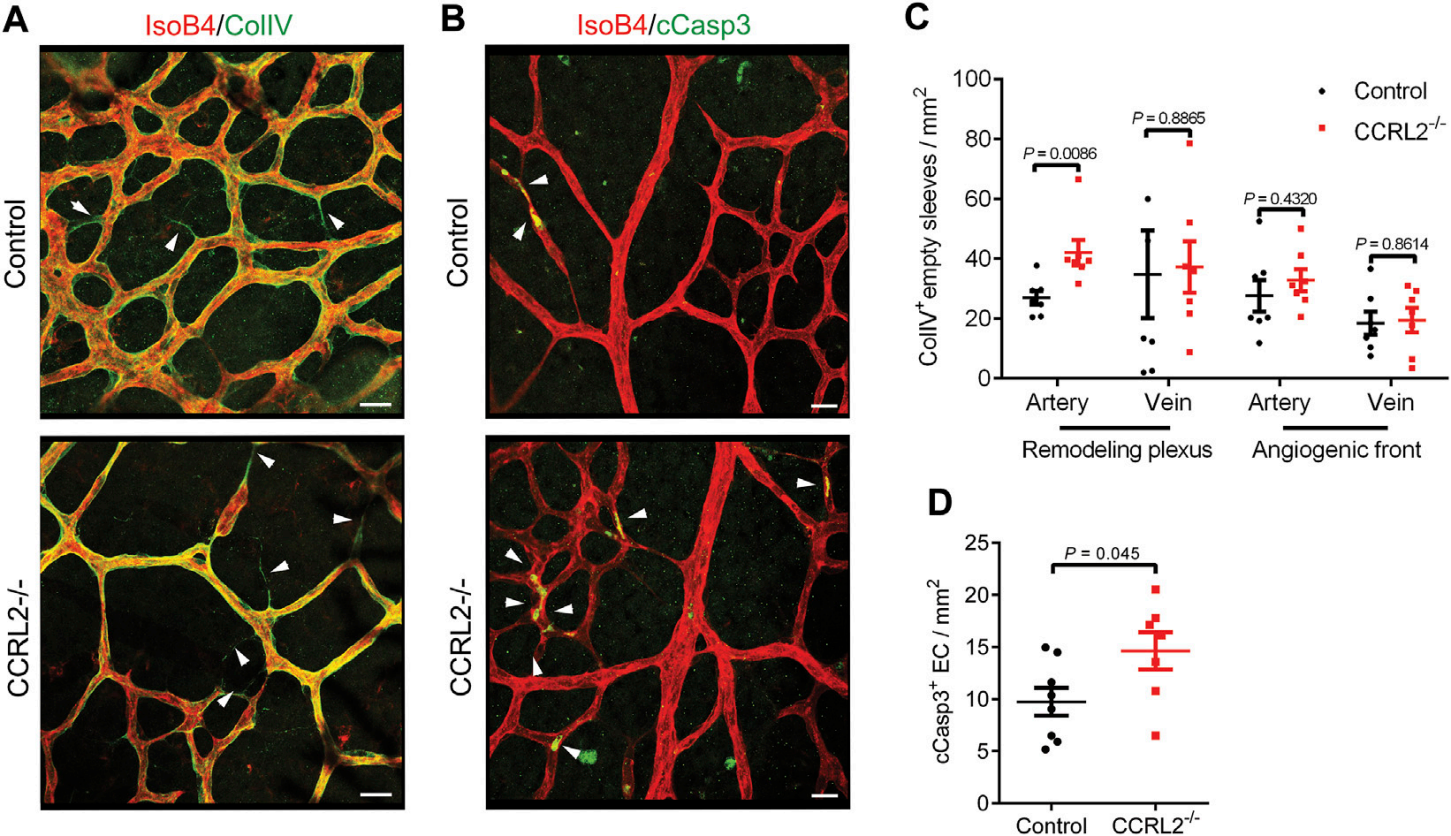
CCRL2 loss-of-function promotes vessel regression. **(A,B)** Confocal images of the remodeling part of the vascular plexus in retinas collected from control or CCRL2^-/-^ mice at P6, stained with IsoB4 and for collagen IV (ColIV, **A**) or with IsoB4 and for cleaved caspase 3 (cCasp3, **B**). ColIV^+^ IsoB4^–^ empty sleeves and apoptotic cells are indicated by white arrows. Scale bars: 20 μm. **(C)** Density of empty sleeves in the remodeling and angiogenic zones of the vascular plexus, respectively around arteries and veins (*n* = 7). Each point represents one retina. **(D)** Density of cCasp3^+^ apoptotic endothelial cells in areas surrounding arteries in the remodeling zone of the plexus from a retinal quadrant (*n* = 8 for controls and 7 for CCRL2^-/-^). Mean ± SEM, 2-tailed unpaired Student’s t test.

The normal development of the vascular network in the retina of newborn mice involves a phase of regression, during which newly formed blood vessels, generated in excess during the sprouting phase, are removed by a process involving vessel constriction, as well as endothelial cell migration and apoptosis. Vessels undergoing regression are seen as empty sleeves after staining of their basal lamina for type IV collagen (ColIV) (Korn and Augustin, 2015). In the remodeling zone of P6 retinas from CCRL2^-/-^ mice, empty sleeves were significantly more numerous, particularly around arteries (Figures 2A,B, arrowheads), while no differences were found around veins in the remodeling plexus, or close to the angiogenic front of the network (Figure 2C). Apoptotic cells, stained for cleaved caspase 3, were also more abundant in the remodeling zone of retinas from P6 CCRL2^-/-^ mice (Figures 2B,D).

### The Retinal Phenotype Is Maintained in Adult CCRL2^-/-^ Mice and Involves CMKLR1

**FIGURE 3:**
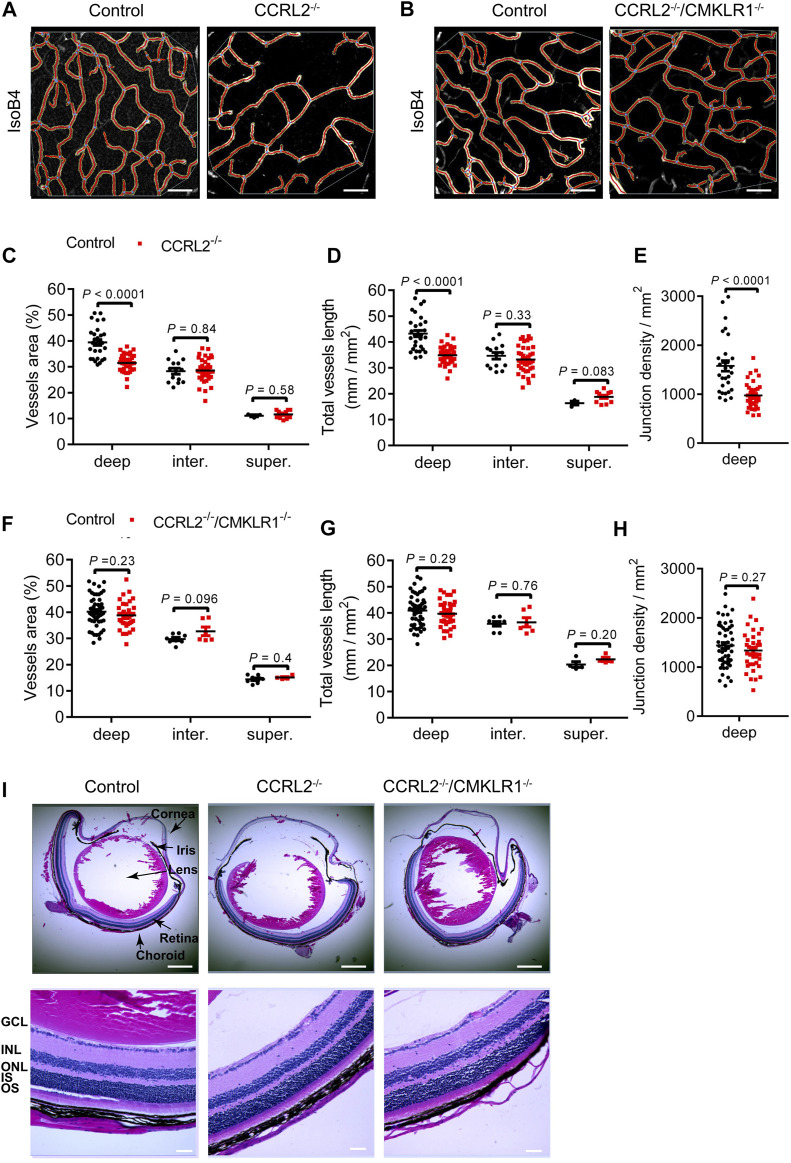
The low density of the vascular network persists in the retina of adult CCRL2^-/-^ mice, and is CMKLR1-dependent. **(A,B)** Deep layer of the vascular network from adult control, CCRL2^-/-^ and CCRL2^-/-^/CMKLR1^-/-^ retinas, stained with IsoB4. Scale bars: 50 μm. **(C–E)** Vessels area, total vessels length and number of junctions (normalized for the surface) in the deep (*n* = 29 for controls, *n* = 38 for CCRL2^-/-^), intermediate (*n* = 14 for controls, *n* = 40 for CCRL2^-/-^) and superficial layers (*n* = 3 for controls, *n* = 12 for CCRL2^-/-^) of the vascular plexus from adult mouse retinas. **(F–H)** Vessels area, total vessels length and junctions density in the deep (*n* = 52 for controls, *n* = 37 for CCRL2^-/-^/CMKLR1^-/-^), intermediate (*n* = 8 for controls, *n* = 6 for CCRL2^-/-^/CMKLR1^-/-^) and superficial layers (*n* = 8 for controls, *n* = 4 for CCRL2^-/-^/CMKLR1^-/-^) of the retinal vascular plexus from WT or CCRL2^-/-^/CMKLR1^-/-^ adult mice. **(I)** H&E-stained sagittal sections of eyes from 8-weeks-old control, CCRL2^-/-^ and CCRL2^-/-^/CMKLR1^-/-^ mice. Scale bars: 500 μm in upper panels, 50 μm in lower panels. GCL, ganglion cell layer; INL, inner nuclear layer; IS, inner segment, ONL, outer nuclear layer; OS, outer segment. Mean ± SEM, 2-tailed unpaired Student’s t test. Each point represents one field of view.

Changes observed during development of the retinal vascular network are frequently compensated later. We therefore analyzed the parameters of the vascular network in the mature retina of adult mice aged 6–8 weeks. The vessel density was found to be lower in the deep layer of the retinal network in adult CCRL2^-/-^ mice, as compared to WT littermates, while no changes were observed for the intermediate and superficial layers (Figures 3A,C–E). The morphology of vessels in the deep plexus appeared also different in CCRL2^-/-^ mice. They were more tortuous than the vessels of control mice. Vessels connecting the intermediate and deep layers were also less organized. These observations, which mimic what was seen previously in chemerin-overexpressing mice (Ben Dhaou et al., 2021) show that no compensatory mechanisms counteract the consequences of CCRL2 loss-of-function, after the initial stages of retinal development.

CMKLR1 is the main functional receptor of chemerin. To determine the contribution of CMKLR1 in the phenotype of CCRL2^-/-^ mice, we analyzed the retina of mice knocked out for both receptors. The vessels area, total vessels length, and junctions density were not modified in adult CCRL2^-/-^/CMKLR1^-/-^ mice compared to WT controls (Figures 3B,F–H), indicating that CMKLR1 is involved in the retinal phenotype of CCRL2^-/-^ mice. The histological aspect of retinas of adult CCRL2^-/-^ and CCRL2^-/-^/CMKLR1^-/-^ mice and their various cell layers were otherwise normal on sections stained by hematoxylin and eosin (Figure 3I).

### CCRL2 Loss-Of-Function Protects From Aberrant Angiogenesis in the Oxygen-Induced Retinopathy Model

**FIGURE 4:**
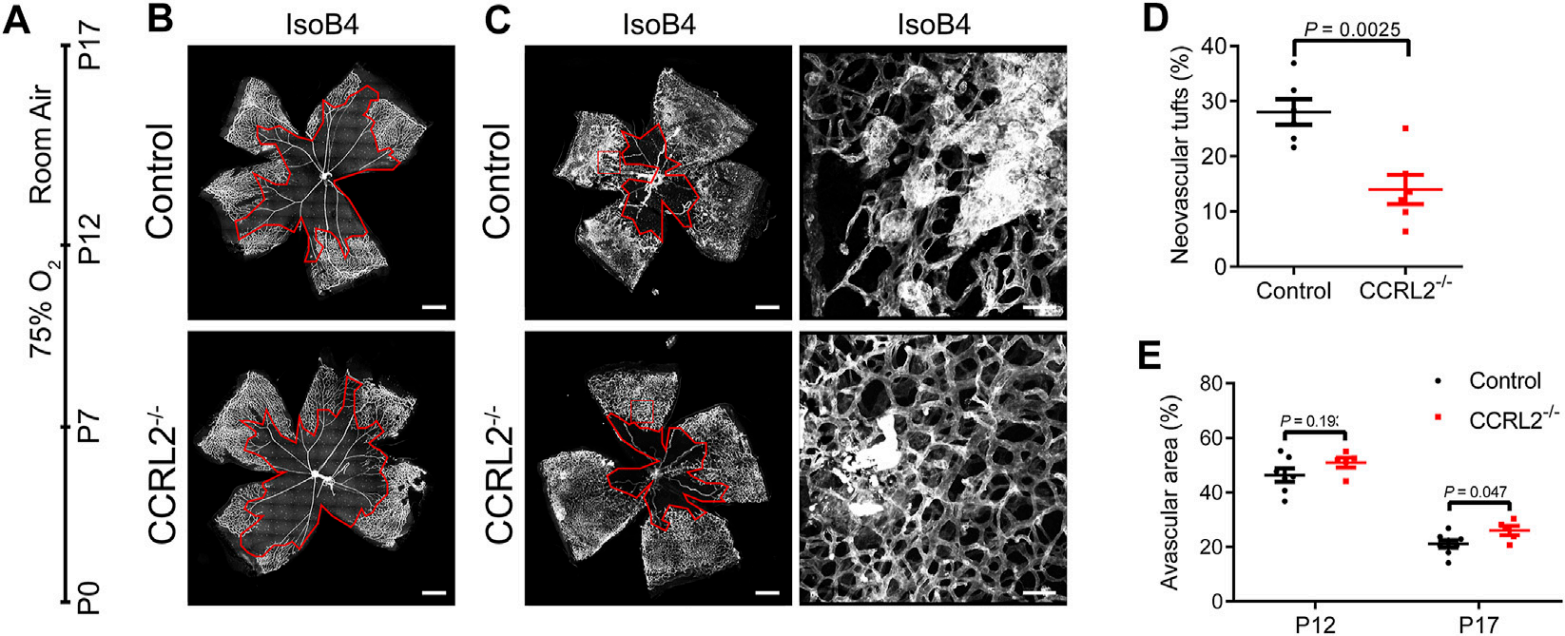
CCRL2 loss-of-function protects against aberrant neovascularization in the oxygen-induced retinopathy (OIR) model. **(A)** Experimental timeline of the OIR protocol, in which mouse pups are exposed to 75% oxygen from P7 to P12, then to normoxia until P17. **(B,C)** Retinal vascular network of control and CCRL2^-/-^ mice stained with IsoB4 at P12 and P17. Scale bars: 500 μm in left panels, 50 μm in right panels. The avascular area is surrounded by the red line. **(D)** Relative area of vascular tufts in control and CCRL2^-/-^ mice (*n* = 6). **(E)** Relative avascular area in control and CCRL2^-/-^ mice at P12 and P17 (*n* = 7 for P12 controls, *n* = 5 for P12 CCRL2^-/-^, *n* = 8 for P17 controls, *n* = 6 for P17 CCRL2^-/-^). Mean ± SEM, 2-tailed unpaired Student’s t test. Each point represents one animal.

The consequences of CCRL2 loss-of-function on the physiological angiogenesis of retina prompted us to evaluate whether the lack of CCRL2 might be protective in a model of pathological angiogenesis. The mouse oxygen-induced retinopathy (OIR) model is mechanistically very similar to an ocular disease frequent in premature infants, the retinopathy of prematurity (ROP), with a phase of vessel obliteration caused by hyperoxia, followed by the exuberant proliferation of abnormal vessels as a result of hypoxia (Scott and Fruttiger, 2010). Following exposure to 75% oxygen from P7 to P12, mouse pups were returned to normoxic conditions at P12 (Figure 4A) (Smith et al., 1994; Campochiaro and Hackett, 2003). The consequences of hyperoxia were similar in CCRL2^-/-^ and control mice at P12, with a marked regression of blood vessels in the central part of the retina (Figures 4B,E), suggesting that CCRL2 does not contribute significantly to this phase of the model. In contrast, the surface occupied by neovascular tufts, the main component of pathological neovascularization formed as a result of hypoxia (Connor et al., 2009), was considerably larger in the retinas of CCRL2^-/-^ mice at P17, as compared to WT littermates (Figures 4C,D). The retinal surface remaining avascular was also somewhat larger in CCRL2^-/-^ pups at P17 (Figures 4C,E). These observations indicate that CCRL2 loss-of-function is protective against excessive neovascularization in the mouse OIR model, reducing the formation of vascular tufts.

### Chemerin Is Elevated in the Blood of CCRL2^-/-^ Mice

**FIGURE 5:**
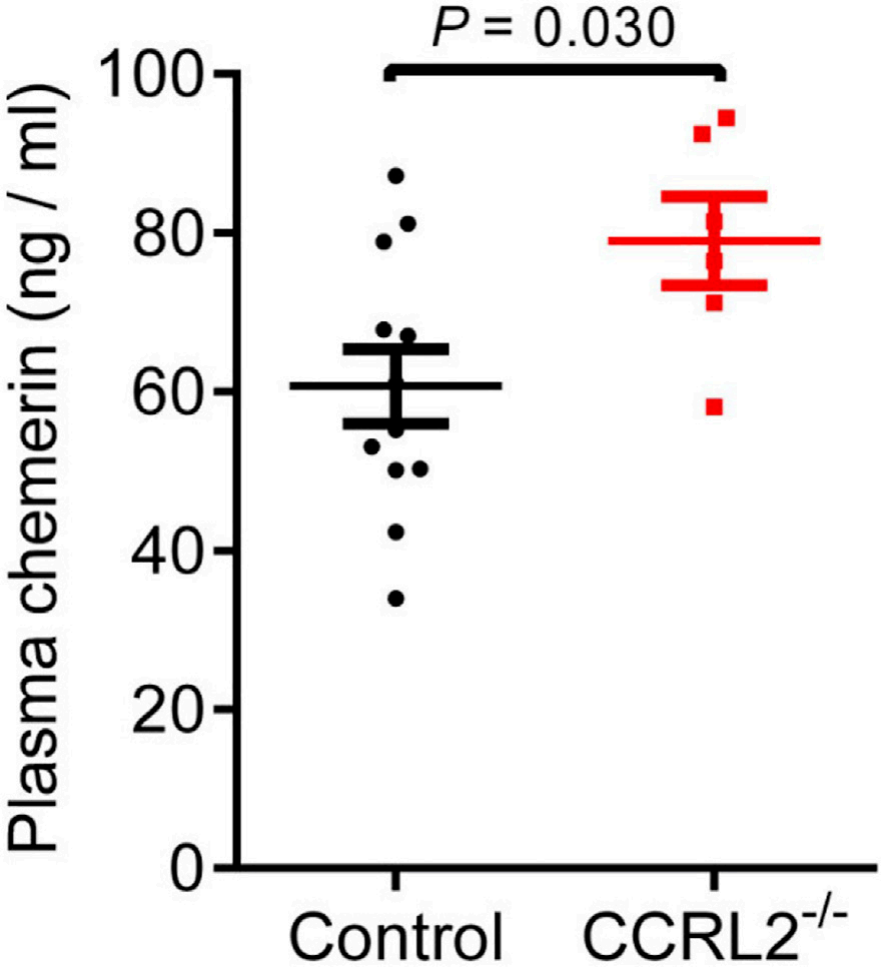
CCRL2 regulates circulating chemerin levels *in vivo*. Chemerin immunoreactivity measured by ELISA in the plasma of control and CCRL2^-/-^ mice at postnatal day 6 (*n* = 12 for controls, *n* = 6 for CCRL2^-/-^). Mean ± SEM, 2-tailed unpaired Student’s t test. Each point represents one animal.

The concentration of chemerin was assayed by ELISA in the plasma of P6 mice, during the development of the vascular network of the retina (Figure 5A). Plasma chemerin levels were significantly increased in CCRL2^-/-^ mice, suggesting that CCRL2 is involved in the regulation of circulating levels of chemerin, thereby controlling its bioactivity in naive conditions.

## Discussion

In previous studies, we demonstrated that chemerin displays anti-angiogenic properties in a number of physiological and pathological settings. We showed that chemerin can inhibit the formation of sprouts in the bead sprouting assay *in vitro* using human endothelial cells (HUVEC) and inhibit the vascularization and growth of tumor grafts in mice (Dubois-Vedrenne et al., 2021). *In vivo* as well, we demonstrated that high chemerin levels in mice can reduce the density of the vascular network in the developing retina, through the pruning of vascular branches and apoptosis of endothelial cells (Ben Dhaou et al., 2021). All these effects were mediated by CMKLR1.

In the present work, we investigated the consequences of CCRL2 loss-of-function on the post-natal development of the vascular network in the mouse retina. This model became over the recent years a classical model of angiogenesis, allowing to study *in vivo* the various steps of the process in a physiological context, with solid and reproducible parameters. We showed that, in the absence of CCRL2, the growth of the vascular network from the center to the periphery of the retina is not affected, while a higher number of vascular branches regressed in the remodeling zone of the plexus, a process accompanied by EC apoptosis. As a result, the density of the vascular network was decreased all along the developmental stages. In other situations affecting early steps in the formation of the vascular network in mouse retina, the changes are compensated at later stages and the adult retina appears unaffected (Ishida et al., 2003; Kubota et al., 2009). The retinas of adult CCRL2^-/-^ mice kept however a lower density in the deep layers of its vascular network, showing that in this case, the developmental change was not compensated later in life.

This phenotype was almost identical to that observed in mice overexpressing bioactive chemerin in basal keratinocytes of the skin and presenting high levels of chemerin in their blood (Ben Dhaou et al., 2021). The retinal phenotype of CCRL2^-/-^ mice was completely reversed in animals knocked-out as well for the CMKLR1 receptor, demonstrating that the anti-angiogenic consequences of CCRL2 loss-of-function involves an hyperactivation of the chemerin/CMKLR1 pathway. In line with this, the chemerin levels were found elevated in the blood of CCRL2^-/-^ mice at post-natal day 6, during the developmental phase of the retinal vascular network. Such increase was also described previously in adult CCRL2^-/-^ mice under naive conditions (Monnier et al., 2012).

We showed previously that chemerin can also temper pathological neoangiogenesis in the oxygen-induced retinopathy model (Ben Dhaou et al., 2021). We observed here, in CCRL2^-/-^ mice, a significant reduction in the formation of vascular tufts as compared to control mice, and a moderate delay in recovering a normal vascular network. This phenotype is also reminiscent of that observed for chemerin-overexpressing mice. CCRL2 can therefore influence pathological angiogenesis as well, playing in this case a detrimental role by dampening the activity of endogenous chemerin.

It should be noted that CCRL2 loss-of-function affects the efficiency of retinal angiogenesis, while CMKLR1 deficiency does not. This means that in the presence of physiological levels of CCRL2 expression, endogenous chemerin does not affect the angiogenesis process, and this can likely be attributed to the buffering activity of CCRL2, and/or its ability to remove the ligand from the circulation and the extracellular medium. CCRL2 might therefore play important roles in controlling the activity of chemerin in both directions. In the context of physiological angiogenesis, CCRL2 protects the network under development from the anti-angiogenic properties of chemerin. In inflammatory situations however, CCRL2 is strongly upregulated in various cell types, including endothelial cells. In this context, the concentration of chemerin bound to CCRL2 might instead increase locally the bioactivity of the ligand, modifying the trafficking of leukocytes but also potentially the angiogenesis process generally associated with inflammatory states.

Since our CCRL2 KO model is constitutive, it is not known whether a specific expression site is responsible for the modulation of the angiogenesis process. CMKLR1 is expressed by endothelial cells and chemerin can directly affect the properties of these cells. Indeed, chemerin inhibited the formation of vessel sprouts by HUVECs in the bead sprouting assay *in vitro*, in the absence of other cell types expressing CMKLR1 (Dubois-Vedrenne et al., 2021). CCRL2 is also expressed by endothelial cells and the endothelium certainly contributes to the regulation of chemerin activities, particularly in the frame of leukocyte trafficking. However, many other cell types expressing CCRL2, including leukocyte subsets, adipocytes and epithelial cells, may also play the role of chemerin sink. Cell-type specific knock out lines might help delineate whether CCRL2 inactivation in one of these cell populations plays a dominant role in the elevation of the circulating chemerin levels that leads to its anti-angiogenic effects.

Chemerin is a chemoattractant factor for leukocyte populations but is structurally unrelated to the large family of chemokines. Two of the chemerin receptors, CMKLR1 and GPR1, are evolutionary closer to receptors for complement peptides (C5a_1_, C3a), formylpeptides (FPR1, FPR2, FPR3) and leucotrienes (BLT_1_, BLT_2_) than to chemokine receptors. CCRL2 however is structurally related to receptors for inflammatory chemokines. Many chemokines and chemokine receptors were described to regulate angiogenesis positively or negatively, in addition to their roles in leukocyte chemoattraction and immunity (Keeley et al., 2011; Bosisio et al., 2014). The new functions attributed to chemerin, CMKLR1 and CCRL2 are therefore reminiscent of the dual activities of several chemokine systems and provide further links between inflammation and angiogenesis. Chemokines encompass two main subfamilies, according to the relative position of the first two cysteines involved in the formation of disulfide bonds, CC and CXC-chemokines (Zlotnik and Yoshie, 2012). The CXC subfamily itself is divided into ELR^+^ and ELR^−^ chemokines following the presence or absence of this amino acid motif in their N-terminal domain. ELR^+^ CXC-chemokines, including CXCL1 to 3, and CXCL5 to 8 are pro-angiogenic. These properties are at least partially mediated by a direct effect on endothelial cells that express their common receptor, CXCR2 (Keeley et al., 2011). These chemokines are indeed able to promote proliferation and migration of endothelial cells *in vitro*. However, part of the angiogenic effects *in vivo* can also be mediated through the recruitment of leukocyte populations, such as neutrophils, which in turn may release cytokines stimulating the production of pro-angiogenic factors through the HIF pathway. Some ELR^−^ CXC-chemokines, including CXCL4 and CXCL9 to 11 display anti-angiogenic properties. These effects may also involve direct effects on endothelial cells as well as indirect effects through the recruitment of leukocyte populations that in turn inhibit angiogenesis (Bosisio et al., 2014). CXCL9, CXCL10 and CXCL11 act through their common receptor CXCR3, which is expressed by endothelial cells. CXCL4 was described to act through CXCR3 as well, but is also affecting endothelial cells in a CXCR3-independent manner.

The ELR^−^ chemokine CXCL12 and its receptors CXCR4 and CXCR7 constitute also a pro-angiogenic system, that in contrast to the other chemokines, contributes to physiological angiogenesis during development. Indeed, CXCL12 or CXCR4 KO mice display developmental defects of the vascular bed in the gut and kidney (Tachibana et al., 1998). Atypical chemokine receptors (ACKR) were also shown to influence angiogenesis by modulating the activity of chemokines and their receptors (Bosisio et al., 2014). In this regard, the chemerin system appears to behave in a very similar way to chemokine systems, controlling the trafficking of specific leukocyte subpopulations, but also modulating angiogenesis, through a direct action on endothelial cells, but also indirectly through the specific effects of each leukocyte subset they recruit. These various activities contribute to the intricate relationships linking inflammation and angiogenesis.

Altogether, our data support the important role of CCRL2 as a modulator of the chemerin/CMLKLR1 axis, and of the effects of this system on inflammation, tumorigenesis and angiogenesis. CMKLR1 agonists are being considered for their potential therapeutic use in some inflammatory diseases and cancer types. However, these strategies should also take into account the role CCRL2 may play on the chemerin/CMKLR1 axis, and the consequences of local CCRL2 expression in this context.

## Data Availability

The raw data supporting the conclusion of this article will be made available by the authors, without undue reservation.
